# The Diagnostic Yield of Implantable Loop Recorders Stratified by Indication: A “Real-World” Single-Center Experience

**DOI:** 10.3390/jcm14041052

**Published:** 2025-02-07

**Authors:** Lorenzo Pistelli, Andrea Di Cori, Matteo Parollo, Marco Torre, Federico Fiorentini, Valentina Barletta, Mario Giannotti Santoro, Gino Grifoni, Antonio Canu, Luca Segreti, Raffaele De Lucia, Stefano Viani, Giulio Zucchelli

**Affiliations:** Department of Cardiac-Thoracic and Vascular, Second Division of Cardiology, Pisa University Hospital, 56124 Pisa, Italy

**Keywords:** implantable loop recorder, syncope, arrhythmia detection, diagnostic yield, real-world data

## Abstract

**Background/Objectives:** Implantable loop recorders (ILRs) are widely used for the diagnosis of unexplained syncope, palpitations, and cryptogenic stroke. While ILRs demonstrate clinical utility, data on their diagnostic yield and value in real-world settings remain limited. This study evaluates ILR performance, diagnostic yield, and clinical impact across multiple indications. **Methods:** We retrospectively analyzed 316 patients who underwent ILR implantation between 2017 and 2023 at a single center. Indications included unexplained syncope, palpitations, and atrial fibrillation (AF) detection. Diagnostic yield, defined as the ratio of positive diagnoses to implants, and diagnostic value, defined as diagnoses leading to therapeutic changes, were assessed. Diagnostic appropriateness, reflecting diagnoses consistent with implant indications, was also investigated. Continuous variables were analyzed using an independent samples *t*-test or ANOVA, when appropriate; dichotomous variables were analyzed using a chi-square test. **Results:** The overall diagnostic yield was 30%, with most diagnoses occurring within 24 months post-implantation. Bradyarrhythmias were diagnosed earlier (mean: 290 days) than tachyarrhythmias (590 days, *p* = 0.04). The diagnostic value was 29%, and the appropriateness reached 70%. The diagnostic-value-to-diagnostic-yield ratio was shown to be as high as 97%, suggesting that whenever a diagnosis was made, it was of clinical impact. Patients with presyncope showed a higher diagnostic yield, particularly for tachyarrhythmias. Device re-implantation showed limited utility, as only one diagnosis (classified as bystander) was achieved in 32 re-implanted patients. After 900 days, the diagnostic yield decreased significantly, with the number needed to follow (NNF) rising from 3.85 to 18 (*p* < 0.001). **Conclusions:** ILRs are effective for arrhythmia detection, demonstrating significant diagnostic and therapeutic impact, particularly within the first two years. The recurrence of presyncope and atrial dilation was associated with higher yields, while isolated syncope posed diagnostic challenges. Prolonged monitoring beyond 900 days and device re-implantation provided diminishing returns.

## 1. Introduction

The implantable loop recorder (ILR) has been a significant advancement in the diagnostic armamentarium available to cardiologists for a variety of clinical syndromes where infrequent arrhythmias are suspected to be the cause of episodic symptoms or when arrhythmic risk stratification is difficult, especially considering that asymptomatic and symptomatic patients share similar outcomes [1,2,3,4,5,6]. Unexplained syncope, palpitations, and cryptogenic stroke (CS) are among the strongest indications [7]. The early use of ILRs in the diagnostic work-up of patients with unexplained syncope or palpitations has been previously established and enables the potential to change therapeutic management and improve quality of life [8,9,10]. Although guidelines recommend the use of ILR in clinical practice, real-world data regarding the diagnostic capability of ILRs in different clinical settings are limited [11,12,13]. Nevertheless, while previous work showed an apparent benefit in terms of the number of diagnoses when extending the ILR monitoring time, whether to extend monitoring time over the lifespan of the ILR (ILR re-implant) is still a matter of debate [14,15,16]. 

The aim of the study was to evaluate the performance of these devices in establishing a diagnosis and their contribution to therapeutic management in a large cohort of unselected patients. Although several previous studies have assessed the diagnostic capability of ILRs, limited data are available on the net clinical impact of the diagnoses made and the extent to which these diagnoses align with the original indication for implantation [17,18]. Furthermore, we sought to explore whether extending the monitoring period beyond the ILR lifespan could be a reasonable approach.

## 2. Materials and Methods

### 2.1. Patient Population, Data Collection, and Definition Adopted

All consecutive patients referred to our center for implantation of an ILR between 2017 and 2023 were retrospectively enrolled in our study. Indications included the investigation of unexplained presyncope/syncope, palpitations, and cryptogenic transient ischemic attack/stroke (CTIA/CS). ILR was considered and implanted when the routine diagnostic work-up had not established a definite diagnosis. All patients with palpitations and unexplained syncope underwent at least a 12-lead ECG, a transthoracic echocardiogram, and 24 h Holter monitoring prior to ILR implantation. Additional diagnostic exams (head-up tilt test, cardiac magnetic resonance imaging, electrophysiology study, etc.) remained at the physicians’ discretion. Cryptogenic TIA and/or stroke were defined as cryptogenic if not attributable to a source of definite cardio embolism, large artery atherosclerosis, or small artery disease. According to the center practice, the diagnostic workflow included at least 24 h Holter monitoring and a transthoracic echocardiogram. The exclusion criteria included high-risk patients, i.e., those with a systolic ventricular dysfunction or a conduction disturbance representing a clear indication for ICD or a pacemaker, or other treatments independent of a definite diagnosis of the cause of symptoms. Informed consent, either written or verbal, was obtained from all patients for their inclusion in the study, and ethical considerations were strictly adhered to. The study protocol aligns with the Helsinki criteria, and formal approval was obtained from the institutional ethics committee, affirming the ethical integrity of the research design and patient participation. 

Data collection included patient information such as age and gender, as well as the specific indication(s) for implantation. Clinical data related to patient cardiovascular comorbidities, as well as CHA2DS2-VASc patient scoring, were also collected. These included a history of hypertension, diabetes, coronary artery disease (CAD), cerebrovascular accident, heart failure (HF), carotid disease, and a history of chronic obstructive pulmonary disease, mitral regurgitation, and hyperlipidemia. ECG, echocardiographic, and device data were systematically reported.

The term “diagnostic yield” was defined as the ratio of positive diagnoses to the total number of ILRs implanted. The term “diagnostic value” was defined as the ratio of positive diagnoses that resulted in changes to clinical practice to the total number of ILRs implanted. The term “diagnostic appropriateness” was defined as the ratio of diagnoses that were consistent with the implantation reason to the total number of diagnoses.

### 2.2. Device Implantation and Remote Control

According to the operator’s discretion, patients received one of the available devices, including Reveal LINQ (Medtronic Inc., Minneapolis, MN, USA), Confirm RX (Abbott Laboratories, Chicago, IL, USA), and Biomonitor III (Biotronik SE & Co. KG, Berlin, Germany), under sterile conditions. Implantations were performed in electrophysiology under local anesthesia and optional antibiotic prophylaxis. As recommended, the insertion tool was positioned at a 45-degree angle at the fourth intercostal space along the left lateral sternum. Patients were discharged the same or the following day.

Device programming included the out-of-box nominal programming recommend by the manufacturer, usually including the criteria of an asystole > 3 s or a primary tachyarrhythmia > 180 bpm lasting 32 Beats. All the implanted ILRs were added to the CareLink or Merlin remote monitoring program with automatic arrhythmia detection programs activated. The automatic alerts by the ILR and the episodes triggered by the patient due to symptoms were evaluated by a technician and adjudicated by a physician.

### 2.3. Follow-Up

Patients were followed both as outpatients every six months and through remote monitoring. Hospital admissions and outpatient visits were retrieved from our electronic database. The last medical contact was considered as the follow-up time. Missing data were excluded from the analyses. Patients lost to follow-up immediately after ILR implantation (e.g., those referred to another hospital for follow-up) were excluded from the study.

### 2.4. Statistical Analysis

Statistical analysis was performed using SPSS software (version 24, IBM Corp., Armonk, NY, USA). Continuous variables were assessed for normality using the Shapiro–Wilk test and subsequently analyzed using an independent samples *t*-test or ANOVA, when appropriate. To assess significant differences over time between two groups, Kaplan–Meier analysis was conducted. Dichotomous variables were analyzed using the chi-square test. In the case of more than two groups, differences between specific groups were highlighted using the standardized residuals test, with Bonferroni correction for multiple-comparison. Cut-off values were defined according to the Youden Index during R.O.C. analysis. Continuous variables are presented as the mean ± standard deviation, while frequencies are expressed as percentages. The level of statistical significance was set at *p* < 0.05, two-tailed.

## 3. Results

### 3.1. Patient Characteristics

A total of 316 patients underwent the implantation of implantable loop recorders (ILRs) at our institution between January 2017 and June 2023. Of these, 55% (175) were male, with a mean age at implantation of 63 ± 17 years. Baseline characteristics did not show statistically significant differences between groups, except for hypertension, which is significantly more frequent among those undergoing an ILR implant for AF (70% vs. 49%, 34%, and 0% in Syncope, Palpitation, and Other groups respectively, *p* = 0.001). 

The primary indications for ILR implantation were unexplained syncope in 58% of cases (183 patients), palpitation in 19% (59 patients), and AF research in 18% (57 patients). Additionally, 5% of patients (17) were monitored for other reasons, such as arrhythmic risk stratification in specific settings with negative EPS but a high suspicion of arrhythmias.

Twenty-five percent (58) of patients underwent further examination (EPS, tilt test, or carotid sinus massage, when appropriate) before the ILR implant. The baseline characteristics of the population in the study are presented in Table 1.

No implant-related complications occurred. During the follow-up, one patient required device removal due to infection after one month. The device did not record any arrhythmias prior to its removal. A new ILR was re-implanted in a different position seven days after the removal. For the purposes of our study, data from both the old and new devices were combined and analyzed as a single device.

Thirty-two (10%) patients underwent device replacement. Among those who replaced the device, only one reached a diagnosis, which was inconsistent with the indication leading to the implant.

### 3.2. Diagnostic Outcomes 

**Figure 1:**
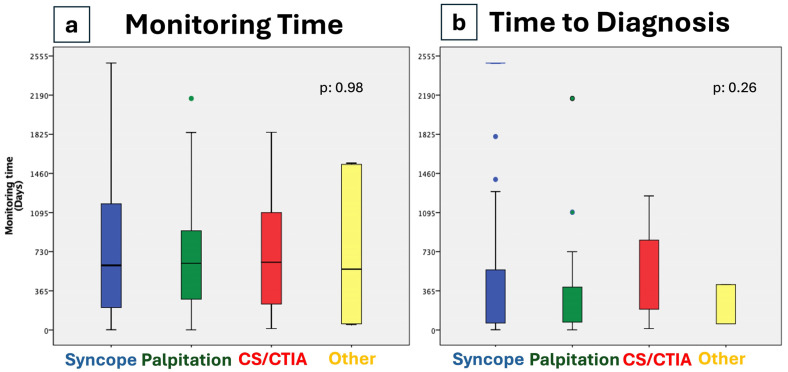
Mean monitoring time (**a**) and time to diagnosis (**b**) according to the indication to implant. CS/CTIA: Cryptogenic Stroke, Cryptogenic Transient Ischemic Attack.

Diagnostic outcomes were observed in 96 patients: 43 (45%) were tachycardia diagnoses and 53 (55%) were bradycardia diagnoses. No statistically significant difference in baseline characteristics was found between patients in which a diagnosis did occur in respect of those in which it did not, except for the symptom of recurrent pre-syncope, which was shown to be more frequent among patients in whom a diagnosis was achieved. The mean monitoring time in patients without diagnosis was 1182 ± 421 days, while when a diagnosis occurred, the mean time to diagnosis was 458 ± 674 (Figure 1, Table 2).

**Figure 2:**
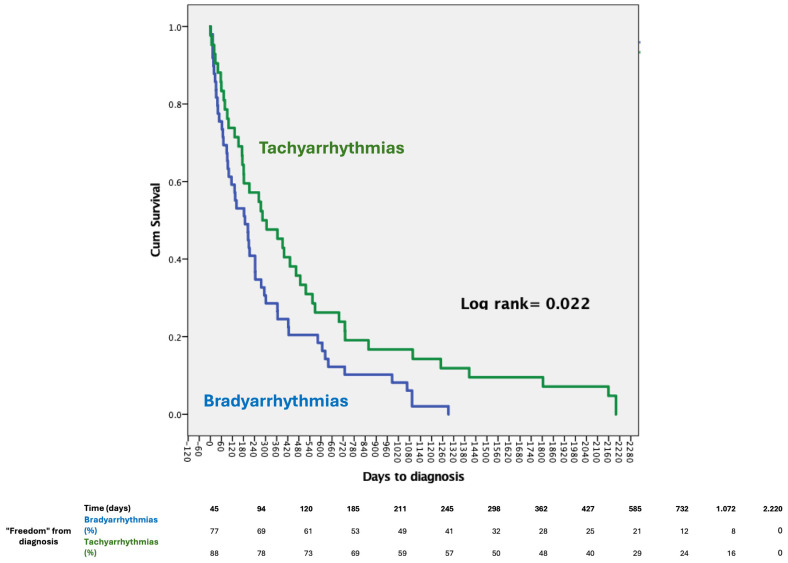
Kaplan–Meier showing different times at diagnosis between bradycardia and tachycardia diagnosis.

**Figure 3:**
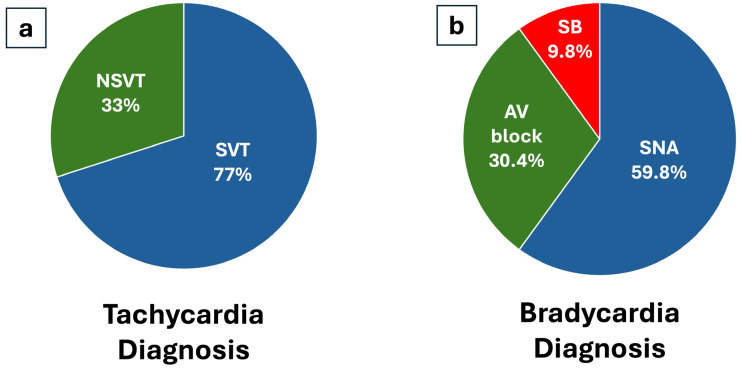
Arrhythmias diagnosed according to Tachyarrhythmias (**a**) and Bradyarrhythmias (**b**) diagnoses. NSVT: Non-Sustained Ventricular Tachycardia, SVT: Supraventricular Tachycardia, AV: Atrioventricular, SNA: Sinus Node Arrest, SB: Sinus Bradycardia.

The diagnosis of bradycardia preceded tachycardia, with mean times of 296 ± 334 days and 529 ± 614 days, respectively (log rank 0.02). Figure 2 illustrates the temporal distribution according to the diagnoses met (Bradycardia or Tachycardia). Table 3 resembles characteristics in tachycardia and bradycardia groups. Most bradycardia episodes were sinus node arrest, with a mean pause of 7.7 ± 5.14 s (Figure 3). Among Tachycardia diagnoses, the most frequent diagnosis was supraventricular tachycardia (77%), followed by non-sustained Ventricular tachycardia (33%) (Figure 3). No episode of sustained VT was recorded.

When considering the subset of patients undergoing ILR monitoring for Syncope or recurrent presyncope, bradyarrhythmia was shown to be the most frequent diagnosis and to occur significantly earlier in respect of a tachyarrhythmia diagnosis (290 vs. 590 days, *p* = 0.040) (Table 3 and Table 4). Among these patients, recurrent pre-syncope was more frequently compelled among those experiencing a tachyarrhythmia (Table 3).

### 3.3. Diagnostic Yield

The overall diagnostic yield (number of diagnoses corrected by number of ILR implant) was 30% (96 patients). No statistically significant differences in diagnostic yield were found according to indication to implant (27% vs. 41% vs. 28% vs. 53% in the syncope, palpitation, AF, and Other groups, respectively, *p* = 0.247, Table 4).

**Figure 4:**
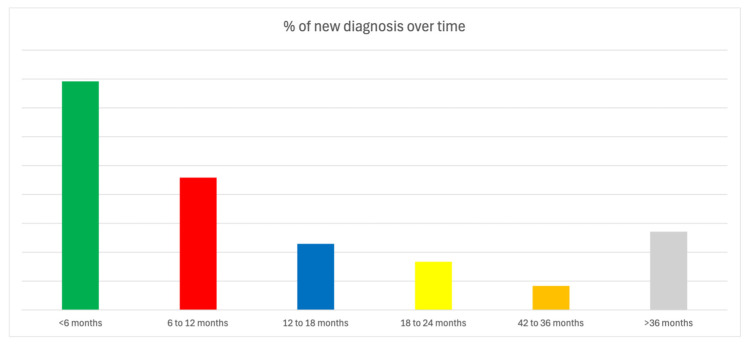
Incidence of new diagnoses (%) over time.

Most diagnoses (86%) occurred within the first 24 months post-implantation. Specifically, 89% of bradycardia diagnoses and 84% of tachycardia diagnoses were made within this timeframe. The diagnostic yield after 900 days was significantly lower compared to that before 900 days; in 14% of cases, a diagnosis was reached after a follow-up longer than 2 years and 6 months (900 days), with a number needed to follow over more than 900 days of 18 to obtain a new diagnosis (vs. 3.85 among population who reached diagnosis within 900 days, *p* < 0.001). Figure 4 shows the incidence of new diagnoses over time.

**Table 4:** Diagnostic Outcomes.

	Overall	Syncope	Palpitation	CS/CTIA	Other	*p*
	316	183	59	57	17	
Diagnosis, *n* (diagnostic yield %)	96 (30)	50 (27)	24 (41)	13 (28)	9 (53)	0.247
Diagnosis tachy, *n* (%)	43 (45)	13 (26)	18 (75)	10 (62)	2 (22)	
Diagnosis brady, *n* (%)	53 (55)	37 (74)	6 (25)	3 (19)	7 (78)	
Diagnostic Value, *n* (%)	93 (29)	50 (27)	21 (36)	13 (28)	9 (53)	
CA, *n* (%)	18 (19)	7 (14)	7 (29)	4 (31)	0 (0)	
Repeted EPS, *n* (%)	9 (9)	4 (8)	3 (13)	1 (8)	1 (11)	
PM implant, *n* (%)	56 (58)	38 (76)	7 (29)	4 (31)	8 (89)	
ICD implant, *n* (%)	5 (5)	1 (2)	4 (17)	0 (0)	0 (0)	
Only medical therapy changes, *n* (%)	5 (5)	0 (0)	0 (0)	4 (31)	0 (0)	
No changes in management, *n* (%)	3 (3)	0 (0)	3 (13)	0 (0)	0 (0)	
Diagnostic value/yield ratio, %	97	100	89	100	100	
Appropriate diagnosis, *n* (diagnostic appropriateness %)	67 (70)	40 (80) *	15 (63)	10 (77) *	2 (22) *	<0.001
Substitution, *n* (%)	32 (10)	16 (9)	10 (17)	5 (9)	1 (6)	
Removal without Diagnosis, *n* (%)	130 (41)	79 (43)	35 (59)	12 (21)	4 (24)	
Time to diagnosis, days (±SD)	458 (674)	440 (641)	490 (904)	455 (568)	490 (421)	0.263
Time to removal (no diagnosis), days (±SD)	1182 (421)	1232 (383)	1163 (413)	864 (673)	1136 (598)	0.378
Mean monitoring time, days (±SD)	831 (606)	765 (582)	1005 (598)	776 (643)	1075 (766)	0.98

CS/CTIA: Cryptogenic Stroke, Cryptogenic Transient Ischemic Attack, CA: Catheter Ablation, EPS: Electrophysiological Study, PM: Pacemaker, ICD: Implantable Cardioverter–Defibrillator. * Statistical significance.

### 3.4. Diagnostic Value

In 63% (61 cases, 20% of the overall population) of cases, the diagnosis led to PM or ICD implant, while in 29% (27 cases), an arrhythmic event detected by ILR led to Catheter Ablation or EPS. Among patients in which ILR led to a diagnosis, 95% underwent an invasive procedure (Table 3). When accounting also for changes in only medical therapy, the overall diagnostic value was 29% (93 out of 316 cases). The ratio between the diagnostic value and diagnostic yield (number of diagnoses which led to a change in patient management regarding the number of diagnosis) was 97%. In three cases, diagnosis did not result in a change in patient management. All of these cases were in the “palpitation” group.

### 3.5. Diagnostic Appropriateness

**Figure 5:**
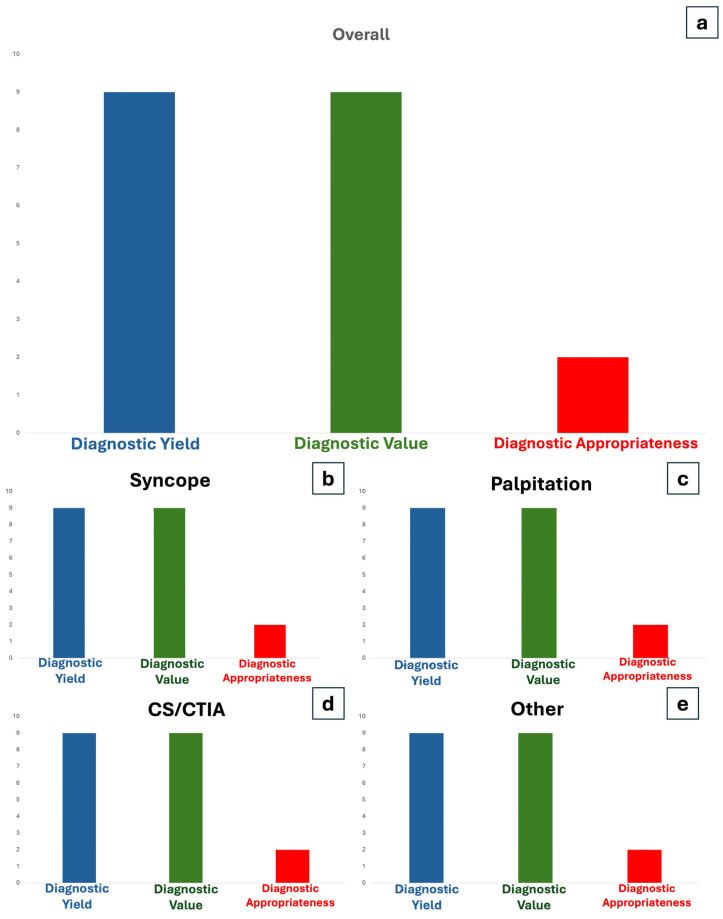
Diagnostic Yield, Value, and Appropriateness, overall (**a**) and according to specific indications to implant (**b**–**e**). CS/CTIA: Cryptogenic Stroke, Cryptogenic Transient Ischemic Attack.

**Figure 6:**
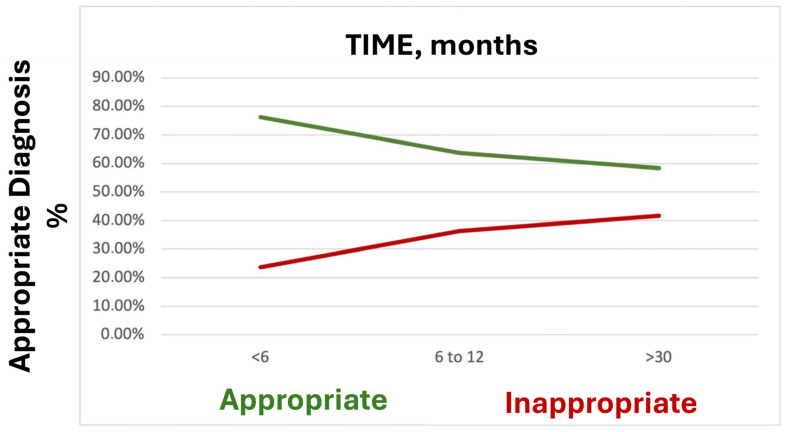
Incidences of appropriate and inappropriate diagnosis over time.

Twenty-one percent of implanted patients received a diagnosis consistent with the initial indication for implantation, accounting for an overall appropriateness of 70% (67 out of 96 diagnoses). Statistically significant differences between groups were observed for the appropriateness of diagnosis (*p* < 0.001), indicating that the “other” group was more frequently associated with a “bystander” diagnosis (78%) compared to Syncope (22%) and AF research (23%), but not Palpitation (Figure 5 resembles the diagnostic yield, value, and appropriateness, overall and according to a specific indication). Although there was a decreasing trend in overall appropriateness over the monitoring time (Figure 6), this trend did not reach statistical significance.

## 4. Discussion

### 4.1. Bradycardia vs. Tachycardia Diagnosis

No independent predictors of diagnosis were identified. However, presyncope was associated with a higher diagnostic yield, particularly for tachyarrhythmic events. This association may be explained by the recurrent nature of presyncope. A symptom that recurs is inherently more likely to recur during monitoring, thereby increasing the chances of detection. Furthermore, in our population, tachyarrhythmic events were either of supraventricular origin or non-sustained ventricular tachycardia. This may justify the better hemodynamic tolerance of these episodes compared to bradyarrhythmias, which, in our cohort, were more frequently associated with sinus arrest (and asystole). Bradyarrhythmias are, therefore, more likely to result in a complete loss of consciousness rather than presyncope.

Notably, in the general population, it is estimated that approximately 30% of syncopal events are isolated episodes without recurrence, further complicating the diagnostic process in such cases [18].

### 4.2. Diagnostic Yield, Diagnostic Value, and Diagnostic Appropriateness

We demonstrated that, in real-world data, the diagnostic yield of ILRs—defined as the number of diagnoses normalized by the number of patients followed—is approximately 30%. Our findings confirm, on a larger scale, the real-world data reported by Smith et al. and are consistent with observations presented by Letsas et al. in a recent large observational study [17,19].

However, except for syncope, certain differences between our study and other similar reports in the literature emerge when focusing on specific indications.

For ILRs implanted for palpitations, Smith and Letsas reported diagnostic yields of 59% and 20%, respectively [17,18]. This variability may likely be attributable to differences in patient susceptibility and clinician sensitivity in attributing clinical relevance to arrhythmias detected by ILRs—such as extrasystoles or short-duration supraventricular tachycardias (SVTs). Our study presents intermediate results between these two extremes, reflecting the high variability of ILR diagnostic yield in this context.

For ILRs implanted to detect atrial fibrillation (AF), we report a higher diagnostic yield compared to previous studies (28% vs. 16% and 19.5% reported by Smith and Letsas, respectively) [17,19]. Nevertheless, as discussed in greater detail below, 23% of ILR-detected diagnoses in this context were classified as “bystander diagnoses”, and AF was identified in 10 out of 57 patients (18%), aligning with data from Smith and Letsas [17,19].

By contrast, Noubiap et al. reported a lower diagnostic yield (10%) in this context. As the authors themselves acknowledged, this difference may partly reflect variations in diagnostic criteria, as their study used a cut-off of 120 s, whereas our center and previous studies applied a 30 s threshold [17,19,20].

**Figure 7:**
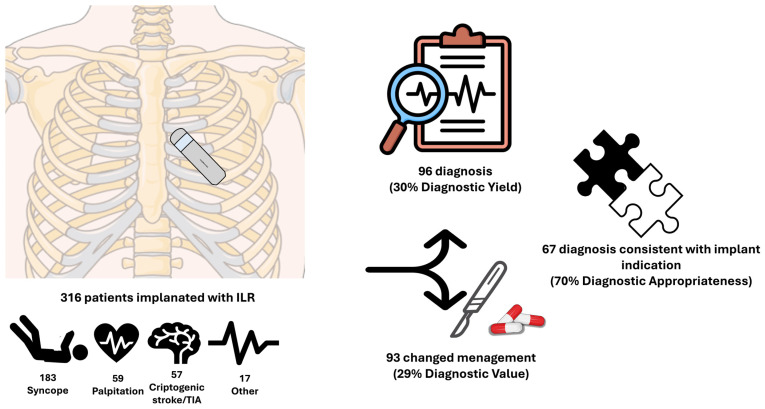
Central Illustration: Diagnostic Yield, Value, and Appropriateness in the population studied.

A distinctive feature of our study was its focus on the diagnostic value of ILRs. Rather than concentrating solely on the identification of arrhythmias, we specifically analyzed clinically significant changes in patient management—including modifications to medical therapy, interventional procedures, or further invasive diagnostic testing—to assess the clinical relevance of the diagnoses made. The diagnostic value of ILRs in our study was 29%, with a diagnostic value-to-diagnostic yield ratio as high as 97%. This high ratio underscores the fact that, when a diagnosis is established, it is frequently clinically impactful. (Central Illustration in Figure 7).

Notably, all cases in which a diagnosis did not lead to substantial changes in clinical management occurred in the “palpitation” group. This finding likely reflects the association between palpitations and clinically insignificant arrhythmias—such as brief episodes of PSVT or extrasystoles—in patients who were not suitable for adjustments in drug therapy, as previously discussed.

According to our data, 28% of patients who received a diagnosis subsequently underwent catheter ablation or EPS following ILR-guided findings. Overall, more than 90% of patients with a diagnosis required some form of interventional treatment, while only 5% (5 patients) underwent changes in medical therapy without requiring further invasive procedures. These findings further highlight the strong clinical impact of ILR-guided diagnoses in our population.

Device implantation rates following ILR diagnosis in our study are consistent with those reported in similar investigations, reinforcing the reliability of our data [19]. However, when focusing on specific indications, some differences emerged. In our opinion, this variability may be partly attributed to the presence of bystander diagnoses and the longer follow-up period in our study.

ILRs, by providing a comprehensive arrhythmic evaluation, inherently carry the risk of detecting bystander diagnoses. We assessed the burden of bystander diagnoses by evaluating diagnostic appropriateness and found an appropriateness rate of 70%, which is slightly lower than that reported by Smith et al. [17].

Two key considerations should be highlighted when interpreting these results. First, defining a diagnosis as a “bystander” is often subjective and inherently difficult to standardize. Second, our data show that the incidence of bystander diagnoses increases with longer monitoring periods. This observation has a logical explanation and may, in part, justify the discrepancy between our results and those reported by Smith, particularly given the differences in the mean monitoring duration.

As it is challenging to implement strategies to limit bystander diagnoses in clinical practice, they should serve as a caution for physicians. Clinicians should carefully consider the likelihood of bystander diagnoses when indicating ILR implantation. 

This aspect becomes even more relevant when examined in light of our finding of a significant increase in the number of patients needed to follow (NNF) to reach a new diagnosis after 900 days. We demonstrated that within the first 900 days of monitoring, the NNF to reach a diagnosis was less than 4, whereas after 900 days, it rose to 18 (*p* < 0.001). This aligns with previous findings in the literature, where most diagnoses were achieved within the first year.

The decline in the incidence of new diagnoses over time could likely be explained by the recurrent nature of most clinically impactful conditions (e.g., conduction block, AF), which are prone to reoccur within a relatively short timeframe in predisposed patients.

We specifically selected a 900-day cut-off to reflect more than half of the typical ILR lifespan. Our data suggest that diagnoses made beyond 2.5 years are relatively rare and offer limited clinical benefit with regard to ILR re-implantation. Nevertheless, in our population, among 32 patients receiving an ILR replacement, only one received a diagnosis (which, notably, was classified as a bystander).

## 5. Conclusions

Implantable loop recorders have proven to be valuable diagnostic tools for specific indications, with a 30% diagnostic yield. Definite diagnoses included both bradyarrhythmic and tachyarrhythmic events, even if the identification of reliable predictors of diagnosis remains challenging. Recurrent presyncope and atrial dilatation recurrence emerged as more effective predictors for tachyarrhythmias, compared to isolated syncope.

The majority of diagnoses were achieved within the first two years post-ILR implantation, suggesting that prolonged monitoring periods may yield diminishing returns in terms of diagnostic value. Finally, device replacement did not demonstrate any significant impact on diagnostic appropriateness. 

Based on our results, ILR re-implantation should be discouraged in routine clinical practice and reserved only for highly selected patients who require specialized monitoring for specific reasons, rather than for the purpose of new diagnoses.

## Figures and Tables

**Table 1:** Baseline characteristics of the population in the study (Overall and according to different groups).

	Overall	Syncope	Palpitation	CS/CTIA	Other	*p*
	316 (100)	183 (58)	59 (19)	57 (18)	17 (5)	
Male, *n* (%)	175 (55)	107 (59)	27 (46)	30 (53)	11 (65)	0.297
Age, y (±SD)	63 (17)	63 (17)	63 (16)	65 (15)	56 (18)	0.35
Weight, kg (±SD)	74 (17)	74 (14)	75 (22)	78 (20)	69 (6)	0.511
Height, cm (±SD)	170 (10)	170 (10)	169 (10)	169 (10)	172 (7)	0.945
BMI, kg/mq (±SD)	26 (4)	25 (4)	26 (6)	27 (5)	23 (2)	0.167
BSA, mq (±SD)	1.8 (0.2)	1.8 (0.2)	1.8 (0.3)	1.9 (0.3)	1.8 (0.1)	0.781
Hypertension, *n* (%)	157 (50)	91 (59)	20 (34)	40 (71) *	0 (0)	0.001
Diabete Mellitus, *n* (%)	36 (11)	22 (50)	4 (7)	10 (18)	0 (0)	0.145
Previous ictus/TIA, *n* (%)	78 (25)	15 (12)	5 (9)	57 (100) *	1 (6)	<0.001
Smoke, *n* (%)	32 (10)	20 (8)	5 (9)	6 (11)	1 (6)	0.902
CAD, *n* (%)	28 (9)	21 (5)	6 (10)	1 (2)	0 (0)	0.64
PAD, *n* (%)	21 (7)	14 (12)	7 (12)	0 (0)	0 (0)	0.415
CHADS VASC, *n* (%)						
0	41 (13)	24 (8)	9 (15)	0 (0)	0 (0)	0.137
1	69 (22)	39 (13)	14 (24)	0 (0)	4 (24)	
2	83 (26)	45 (21)	15 (25)	24 (42)	4 (24)	
3	76 (24)	45 (25)	18 (31)	16 (28)	1 (6)	
4	33 (10)	22 (12)	2 (3)	10 (18)	2 (12)	
5	13 (4)	7 (4)	1 (2)	7 (12)	0 (0)	
6	1 (0.3)	1 (1)	0 (0)	0 (0)	0 (0)	
COPD, *n* (%)	7 (2)	5 (3)	2 (4)	0 (0)	0 (0)	0.953
Serum creatinine, mg/dL (±SD)	1.3 (6)	0.9 (0.2)	0.9 (0.2)	3.5 (0.2)	0.9 (0.2)	0.164
ECHO						
LVEF < 55%, *n* (%)	10 (3)	8 (11)	1 (2)	1 (2)	0 (0)	0.545
LAD, mm (±SD)	40 (8)	40 (9)	37 (7)	41 (6)	45 (12)	0.228
LAV, mL (±SD)	65 (22)	66 (21)	59 (22)	68 (20)	66 (31)	0.682
LAVi, mL/mq (±SD)	34 (12)	36 (13)	29 (10)	37 (11)	36 (14)	0.163
LVEDD, mm (±SD)	48 (5)	48 (6)	47 (5)	49 (5)	47 (3)	0.755
LVEF, % (±SD)	60 (8)	61 (8)	59 (7)	56 (10)	60 (8)	0.207
ECG						
HR, bpm (±SD)	65 (15)	64 (14)	63 (13)	68 (14)	60 (24)	0.541
Sinus rhythm, *n* (%)	294 (93)	179 (98)	90 (153)	19 (33)	6 (35)	0.144
AF/AFl, *n* (%)	16 (5)	9 (5)	7 (12)	0 (0)	0 (0)	
Conduction disturbance, *n* (%)	80 (25)	47 (26)	27 (46)	5 (9)	1 (6)	0.89
Pre-implant investigation						
Tilt test	8 (3)	8 (5)	0 (0)	0 (0)	0 (0)	
Carotid sinus massage	11 (4)	10 (6)	1 (2)	0 (0)	0 (0)	
EPS	59 (19)	30 (16)	19 (32)	8 (14)	2 (12)	

CS: Cryptogenic Stroke, CTIA: Cryptogenic Transient Ischemic Attack, BMI: Body Mass Index, BSA: Body Surface Area, CAD: Coronary Artery Disease, PAD: Peripheral Artery Disease, COPD: Chronic Obstructive Pulmonary Disease, LVEF: Left Ventricle Ejection Fraction, LAD: Left Atrial Diameter, LAV: Left Atrial Volume, LAVi: LAV indexed, LVEDD: Left Ventricle End Diastolic Diameter Fraction, HR: Heart Rate, AF: Atrial Fibrillation, AFl: Atrial Flutter, EPS: electrophysiological study. * statistical significance.

**Table 2:** Baseline characteristics according to diagnosis (Met or Not).

	No Diagnosis	Diagnosis	*p*
Total	220	96	
Male, *n* (%)	122 (56)	53 (55)	0.609
Age, y (±SD)	64 (17)	62 (16)	0.501
Weight, kg (±SD)	74 (18)	75 (13)	0.67
Height, cm (±SD)	170 (10)	169 (9)	0.45
BMI, kg/mq (±SD)	25 (5)	26 (4)	0.236
BSA, mq (±SD)	1.8 (0.3)	1.8 (0.2)	0.758
Hypertension, *n* (%)	103 (47)	54 (56)	0.51
Diabete mellitus, *n* (%)	25 (11)	11 (12)	0.871
Previous ictus/TIA, *n* (%)	43 (20)	12 (13)	0.169
Smoke, *n* (%)	20 (9)	12 (13)	0.294
CAD, *n* (%)	20 (9)	8 (8)	0.911
PAD, *n* (%)	17 (7)	4 (9)	0.277
CHADS VASC, *n* (%)			
0	32 (15)	9 (9)	0.694
1	48 (22)	21 (22)	
2	57 (26)	26 (27)	
3	50 (23)	26 (27)	
4	26 (12)	7 (7)	
5	10 (5)	3 (3)	
6	1 (1)	0 (0)	
COPD, *n* (%)	5 (2)	2 (2)	0.316
Syncope, *n* (%)	121 (55)	45 (47)	0.183
Recurrent Presyncope (>1 episodes), *n* (%)	28 (13)	29 (30)	<0.001 *
Palpitation, *n* (%)	51 (23)	30 (31)	0.265
Prodroms, *n* (%)	31 (14)	14 (15)	0.8
Trauma, *n* (%)	30 (14)	18 (19)	0.423
ECHO			
LVEF < 55%, *n* (%)	7 (3)	3 (3)	0.971
LAD, mm (±SD)	41 (8)	39 (8)	0.493
LAV, mL (±SD)	65 (22)	64 (23)	0.878
LAVi, mL/mq (±SD)	33 (11)	35 (13)	0.509
LVEDD, mm (±SD)	49 (7)	47 (4)	0.142
ECG			
Sinus Rhythm, *n* (%)	208 (95)	86 (90)	0.717
AF/AFl, *n* (%)	10 (5)	6 (6)	
Conduction disturbance, *n* (%)	50 (23)	30 (31)	0.044
Pre-implant investigation, *n* (%)			
Tilt Test, *n* (%)	6 (2)	2 (2)	0.729
Carotid sinus massage, *n* (%)	8 (4)	3 (3)	0.814
EPS, *n* (%)	43 (20)	16 (17)	0.430

TIA: Transient Ischemic Attack, BMI: Body Mass Index, BSA: Body Surface Area, CAD: Coronary Artery Disease, PAD: Peripheral Artery Disease, COPD: Chronic Obstructive Pulmonary Disease, LVEF: Left Ventricle Ejection Fraction, LAD: Left Atrial Diameter, LAV: Left Atrial Volume, LAVi: LAV Indexed, LVEDD: Left Ventricle End Diastolic Diameter, AF: Atrial Fibrillation, AFl: Atrial Flutter, EPS: Electrophysiological Study. * Statistical Significance.

**Table 3:** Characteristics of patients implanted for Syncope according to the diagnosis met (Tachycardia or Bradycardia).

	Tachyarrhythmia	Bradyarrhythmia	*p*
Male	7 (54)	25 (68)	0.378
Age, y	64 (16)	66 (16)	0.595
BMI, kg/mq	24 (3)	27 (3)	0.047
BSA, mq	1.7 (0.1)	1.8 (0.2)	0.566
Hypertension	6 (46)	24 (65)	0.193
Diabete mellitus	8 (62)	5 (14)	0.331
Previous ictus/TIA	0 (0)	5 (14)	0.16
Smoke	4 (31)	4 (11)	0.1
CAD	0 (0)	5 (14)	0.156
PAD	0 (0)	4 (11)	0.21
CHADSVASC			
≤2	7 (54)	20 (54)	
>2	6 (46)	17 (11)	
CKD	0	4 (3)	0.586
COPD	0	1 (3)	0.686
Pre-implant investigation			
Tilt Test	1 (8)	1 (3)	0.43
Carotid sinus massage	1 (8)	1 (3)	0.46
EPS	2 (15)	2 (5)	0.254
Symptoms			
Syncope	10 (77)	35 (95)	0.68
Presyncope	11 (85)	8 (22)	0.042 *
Prodromi	2 (15)	10 (27)	0.398
Trauma	2 (15)	16 (43)	0.072
ECHO			
LAD, mm	35 (5)	42 (10)	0.111
LAV, mL	49 (10)	70 (24)	0.095
LAVi, mL/mq	26 (4)	39 (16)	0.101
LVEDD, mm	48 (3)	46 (5)	0.514
LVEF, %	63 (6)	63 (8)	0.99
ECG			
Sinus rhythm	13 (100)	33 (89)	0.283
Time to diagnosis, days	590 (618)	290 (353)	0.04 *

BMI: Body Mass Index, BSA: Body Surface Area, CAD: Coronary Artery Disease, PAD: Peripheral Artery Disease, CKD: Chronic Kidney Disease, COPD: Chronic Obstructive Pulmonary Disease, LVEF: Left Ventricle Ejection Fraction, LAD: Left Atrial Diameter, LAV: Left Atrial Volume, LAVi: LAV Indexed, LVEDD: Left Ventricle End Diastolic Diameter, EPS: Electrophysiological Study. * Statistical significance.

## Data Availability

Data are available from the corresponding author upon reasonable request.
